# Primary Malignant Synovial Tumor in the Mediastinum

**DOI:** 10.7759/cureus.20076

**Published:** 2021-12-01

**Authors:** Shreyas Bellur, Sreekar Balasundaram

**Affiliations:** 1 Department of Cardiothoracic Surgery, St. John’s Medical College Hospital, Bangalore, IND

**Keywords:** surgical resection, exploratory thoracotomy, hydropneumothorax, mediastinum, synovial sarcoma

## Abstract

Primary mediastinal synovial sarcomas constitute a rare subset of mediastinal tumors. The diagnosis is often delayed at the time of presentation impacting the five-year survival rate due to its highly aggressive natural history. We report a 22-year old female with a monophasic variant of the primary mediastinal synovial sarcoma.

A 22-year-old female, with a two-month history of productive cough, fever, breathlessness, was referred to our center in view of persistent right-sided hydropneumothorax despite multiple thoracocenteses. Examination revealed reduced right-sided air entry with a succussion splash. Imaging suggested a well-defined cystic lesion with fluid, air foci, and multiple septations. A provisional diagnosis of a ruptured hydatid cyst was made and exploratory thoracotomy was planned. Intraoperatively, a well-defined cystic lesion with 350 mL of hemorrhagic fluid, densely adherent to the lung and diaphragm, was found. The biopsy revealed a monophasic spindle cell variant of the primary synovial sarcoma. Follow-up positron emission tomography (PET) on postoperative day 20 showed no residual disease and evidence of metastasis. However, the patient was lost to follow up following one cycle of chemotherapy with ifosfamide.

Primary mediastinal synovial sarcomas are aggressive tumors that warrant an early diagnosis for prompt treatment. They usually present with non-specific respiratory symptoms. The gold standard of diagnostic modalities is a molecular panel looking for the translocation - t(X;18)(p11;q11). However, in low-middle income countries, a biopsy may be more practical, as they are cost-effective. The treatment is surgical resection, with combined chemotherapy and radiotherapy if metastases are present.

Our case emphasizes the early detection of this lesion, its mimicry with other lesions, and the impact of early diagnosis.

## Introduction

Primary synovial sarcomas are aggressive malignant tumors that are a subtype of soft tissue sarcomas. However, the term “synovial” is a misnomer as the cells of origin do not belong to the synovium, their histogenesis still being largely unknown [1]. Considerably rare to find in the thorax, with only a few case reports in the literature, these tumors seem to occur in middle age with a slight male preponderance [2].

We present a case of a 22-year-old female with primary mediastinal synovial sarcoma (PMSS) mimicking a hydatid cyst.

## Case presentation

A 22-year-old female, with a two-month history of sudden onset cough with expectoration, breathlessness, and fever, and no known co-morbidities, was referred to our center in view of a non-resolving hydropneumothorax despite multiple thoracocenteses. Sputum was grey, with mucoid consistency. She reported no hemoptysis, loss of weight, and history of tuberculosis. She reported no symptoms of orthopnea, paroxysmal nocturnal dyspnea, palpitations, and chest pain, ruling out cardiac involvement. History was significant for poor sanitation and environmental surroundings.

**Figure 1:**
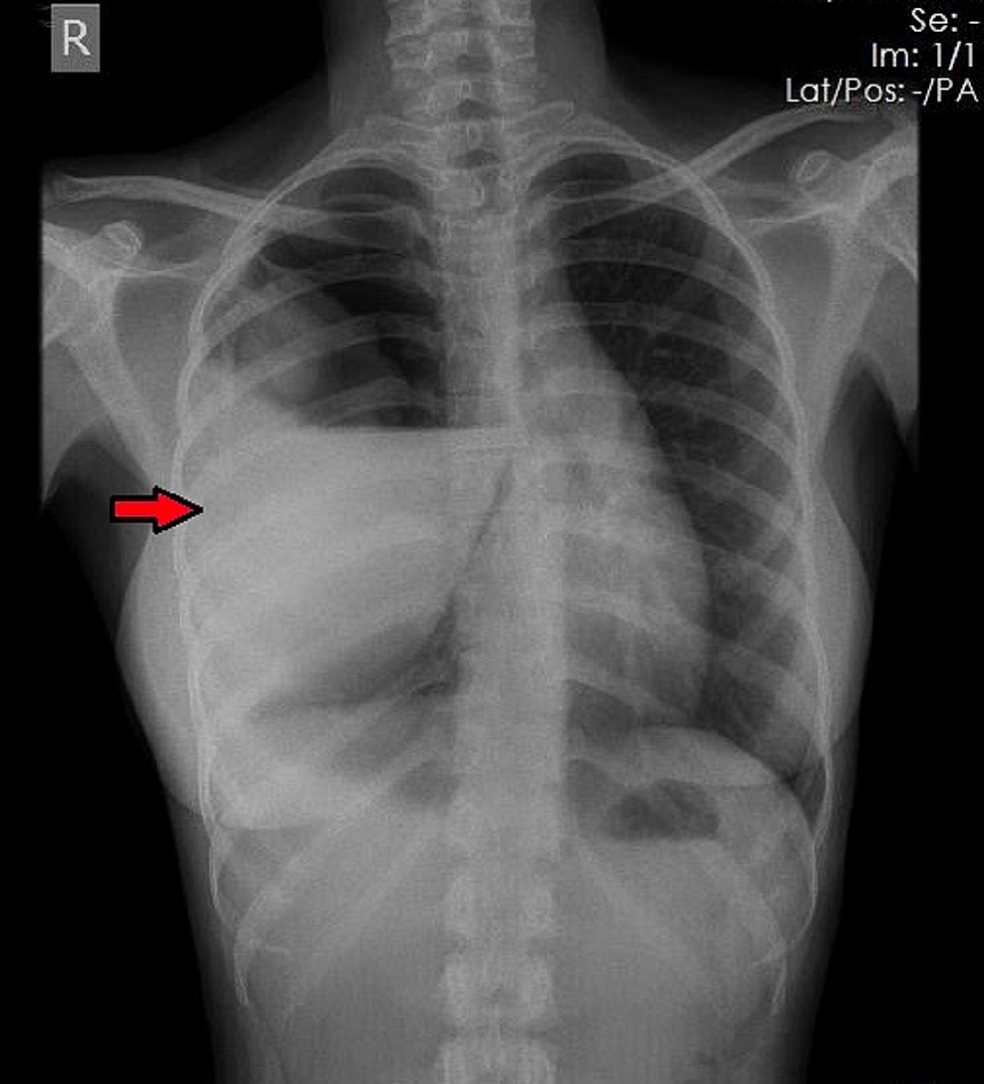
Preoperative chest x-ray showing a right hydropneumothorax

**Figure 2:**
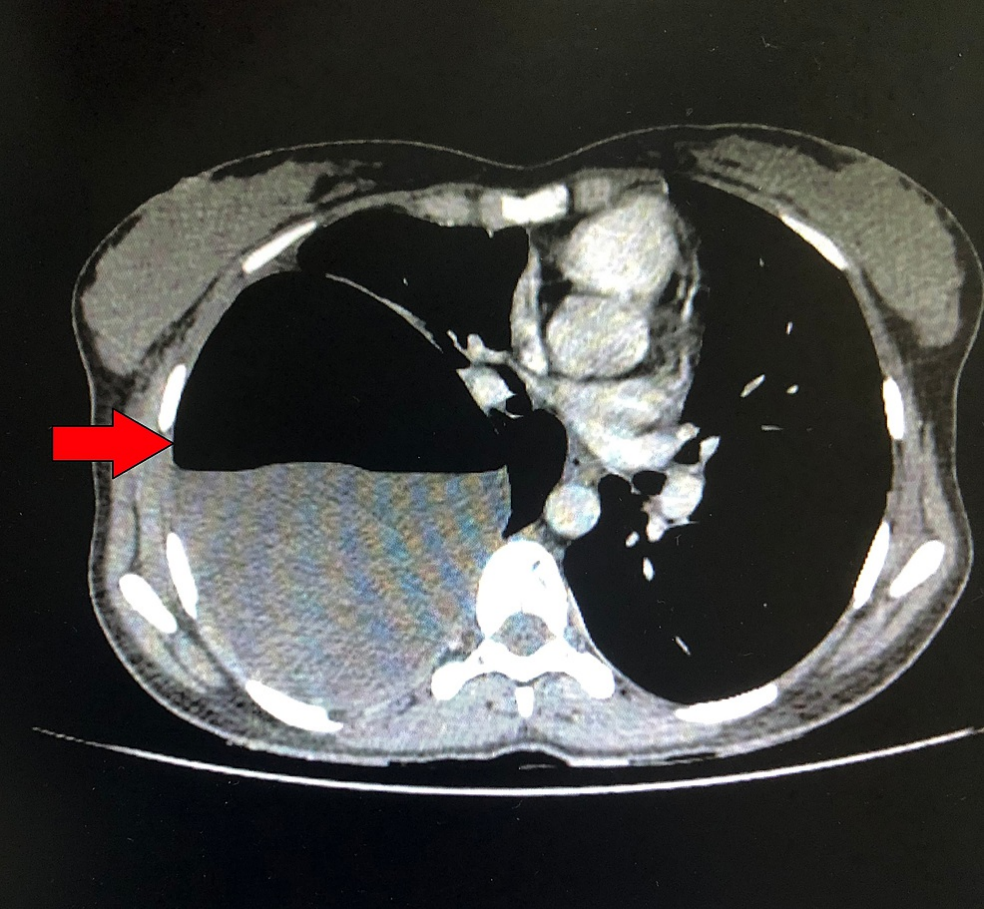
CT chest showing a well-defined fluid-filled cystic lesion in the right hemithorax CT- Computed Tomography

Examination showed reduced air entry on the right side with a succussion splash, indicating a fluid-filled lesion. Other examination findings were unremarkable. Chest X-ray showed right hydropneumothorax (Figure 1) and later computed tomography (CT) of the chest showed a right-sided, well-defined cystic lesion arising from the mediastinum measuring 13 x 11 x 18 cm, with a volume of approximately 500 mL, causing compression atelectasis of the underlying lung and mediastinal shift to the left. Air foci and thin septations were noted with an obvious connection to the airway (Figure 2).

A preoperative diagnosis of a ruptured infected pulmonary hydatid cyst was made and she was counseled regarding the need for surgery - an exploratory thoracotomy. Preoperative investigations were unremarkable. α-fetoprotein and β-human chorionic gonadotropin (β-HCG) were within normal limits. 

**Figure 3:**
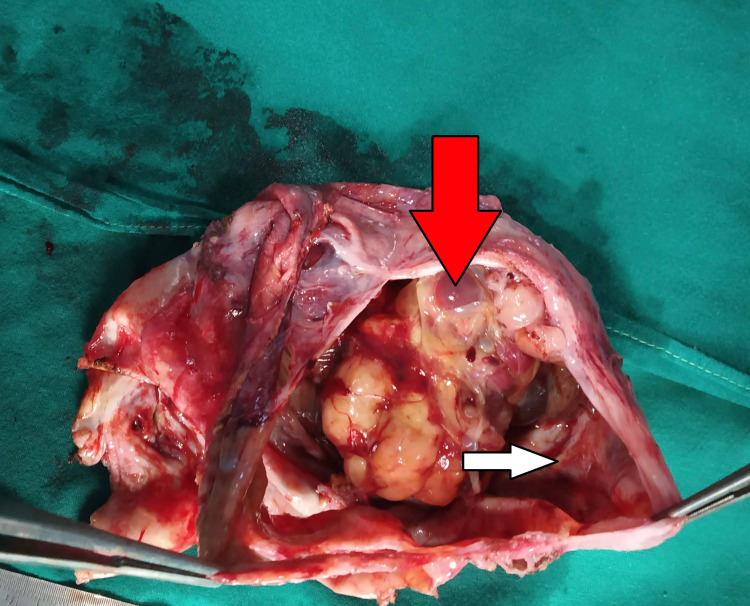
Excised specimen showing solid and cystic areas Red arrow - solid area; white arrow - cystic area

After informed consent, she underwent surgery, and intraoperatively, we found a thick fluid-filled cyst with solid areas and approximately 350 mL of hemorrhagic fluid (Figure 3).

**Figure 4:**
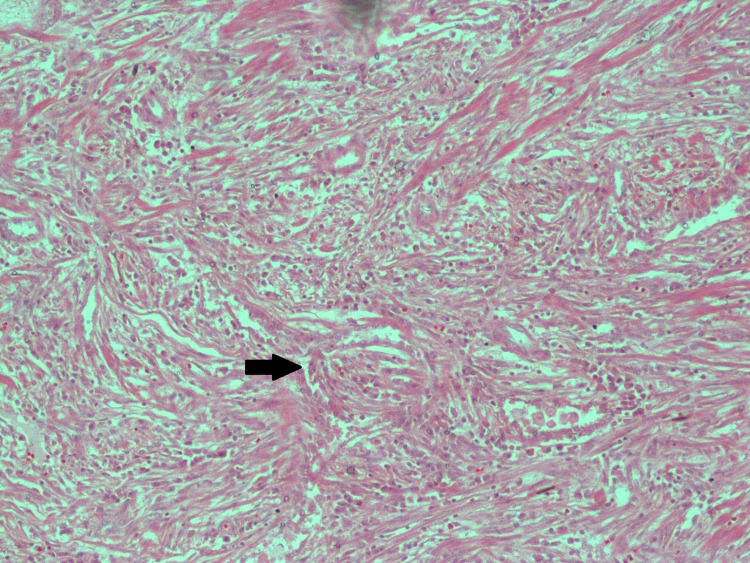
Histopathology specimen showing the monophasic spindle cell variant of the synovial sarcoma Black arrow - Bland spindle cells arranged in fascicles exhibiting oval nuclei and a moderate amount of eosinophilic cytoplasm

There were dense adhesions to the lung and diaphragm and a collapsed right upper lobe. The pleural cyst was excised and sent for biopsy and its contents for fluid analysis. Fluid cytology showed paucicellular cyst fluid with a few lymphocytes and biopsy findings were consistent with monophasic spindle cell variant of the synovial sarcoma (Figure 4).

Immunohistochemistry (IHC) showed membrane positivity for cluster of differentiation 99 (CD99) (3+, >90%), and cytoplasmic positivity for B-cell lymphoma 2 (BCL2) (3+, 90%), cytokeratin (CK) (2+, <5%), epithelial membrane antigen (EMA) (2-3+, <5%) and a proliferative index of 70%-80%.

**Figure 5:**
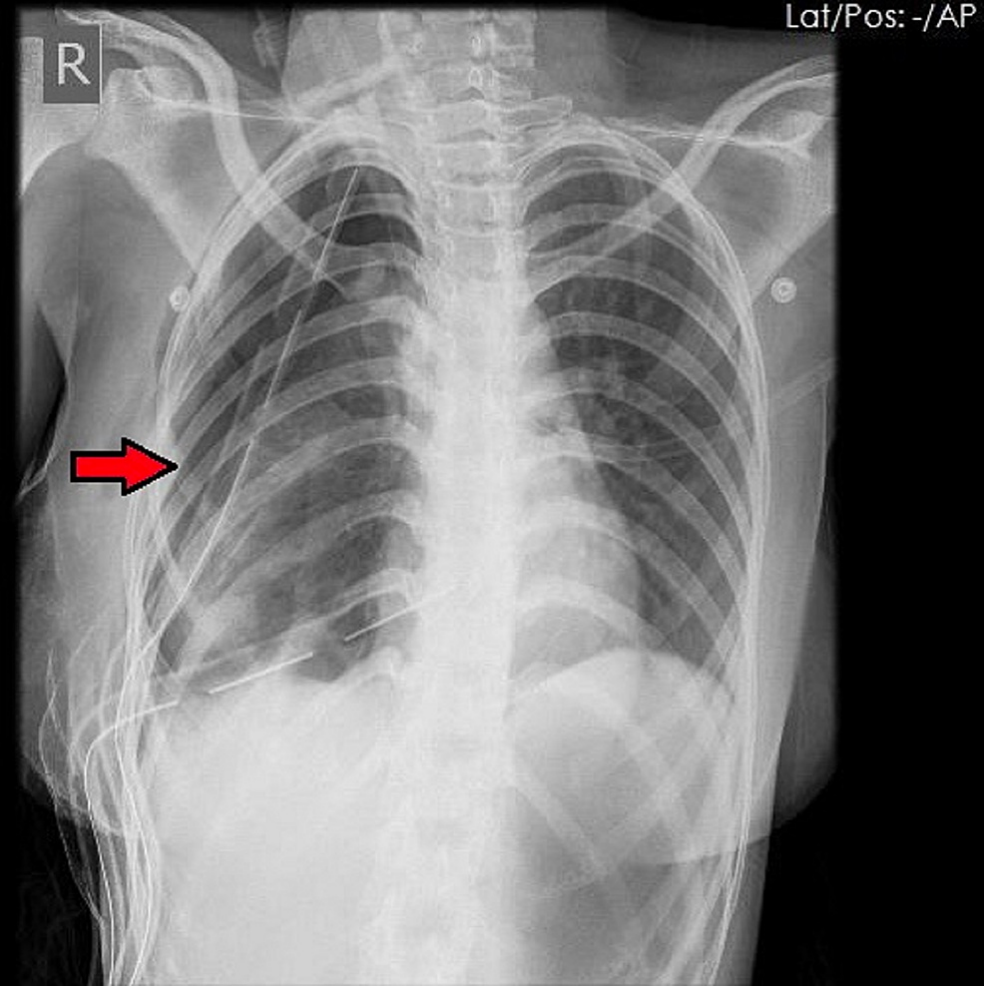
Postoperative chest x-ray showing right lung collapse

Postoperatively, because of the persistent right lung collapse (Figure 5), a bronchoscopy was done and secretions were drained. She was discharged on postoperative day 9 with a right chest tube in situ. The patient was asked to review with medical oncology with a positron emission tomography (PET) report after one week.

On review, she was symptomatically better, postoperative Eastern Cooperative Oncology Group (ECOG) score was 1 (compared to preoperative ECOG score of 2). Chest x-ray showed complete expansion of the right upper lobe, and PET scan showed no evidence of residual disease or metastasis. She was advised chemotherapy, but after one cycle with ifosfamide, was lost to follow up.

## Discussion

PMSSs are extremely rare tumors, with a limited number of case reports in the English literature [3]. These entities are highly aggressive with dire potential for metastasis. They are mostly found in the middle-aged population with slight male preponderance [2].

Erroneously named “synovial sarcomas,” the cellular origin of this neoplasm remains unknown, with mesenchymal stem cells and myoblasts hypothesized to be probable cells of origin [1,4].

The tumor is aptly referred to as a translocation-associated tumor as a translocation t(X;18)(p11;q11) is present in 90% of cases. This translocation gives rise to a fusion protein - SS18-SSX which disrupts gene expression due to aberrant transcription [5]. However, in the remaining 10% of cases, there is no discernable chromosomal abnormality.

At the time of presentation, PMSSs are usually quite big, with symptoms ranging from a productive cough to hemoptysis. An examination is usually significant for reduced air entry and in case of large cystic fluid-filled lesions as in our case, a succussion splash can be elicited. Our case presented findings consistent with existing literature. We considered the diagnosis of a cystic lesion, with our first differential being a ruptured hydatid cyst given the history of poor environmental hygiene and the prevalence of the same in India and the second being a loculated empyema, given the history of fever, and productive cough. We also considered Hodgkin’s lymphoma and mixed germ cell tumor due to age incidence [6,7].

The laboratory investigation panel must include α-fetoprotein and β-HCG to rule out germ cell malignancies. X-ray imaging yields a picture of round to oval lobulated mass. However, certain cases, such as ours, can present with hydropneumothorax. CT findings commonly show a well-circumscribed heterogeneously enhancing mass, as in our case [8]. Molecular diagnosis using fluorescent in situ hybridization looking for the translocation and reverse transcriptase-polymerase chain reaction looking for the novel gene segment cement the diagnosis. However, in low-middle income countries, such as India where genetic testing can be either too expensive or inaccessible for the average patient, histopathology (HP) and IHC need to be used as adjuncts to diagnosis [3]. IHC is usually positive for EMA, CK, BCL2, and CD99 [9]. It is of the utmost importance to rule out metastases, as the mode of treatment depends on their presence or absence. We suggest a PET CT when feasible [10]. If an endobronchial extension is present, we suggest an endobronchial biopsy which can hasten the diagnosis, preventing undue treatment delay. Metastases indicate a poor prognosis, with a dismal five-year survival rate [10]. Surgical resection is the treatment of choice and is usually made by performing a thoracotomy. Complete resection is key to drastically increasing the chances of survival.

## Conclusions

Primary mediastinal synovial sarcomas are rare, and difficult to diagnose aggressive malignancies with a significant potential for metastasis and a poor prognosis. Early diagnosis is key and the need to consider it in a differential when a cystic thoracic lesion is warranted. Histopathological and immunohistochemical correlation is advised in cases where a molecular diagnosis is not feasible. Complete surgical resection with adjuvant chemotherapy is the goal of treatment.
